# Preliminary study on metabolite differences between two obesity syndromes based on Q Exactive liquid chromatography–tandem mass spectrometry metabolomics

**DOI:** 10.1371/journal.pone.0331901

**Published:** 2025-09-10

**Authors:** Ye Jing, Xiaoxuan Xu, Yanhong Wang, Xue Qu, Yunlong Guo, Ao Guo, Yulin Dai, Yangyang Liu, Hao Yue

**Affiliations:** 1 Jilin Ginseng Academy, Changchun University of Chinese Medicine, Changchun, China; 2 Department of College of Traditional Chinese Medicine, Changchun University of Chinese Medicine, Changchun, China; 3 Changchun University of Chinese Medicine, Changchun, China; 4 Department of Prevention and treatment of disease Care Center, The Affiliated Hospital to Changchun University of Chinese Medicine, Changchun, China; Tecnologico de Monterrey, MEXICO

## Abstract

**Background:**

According to traditional Chinese medicine, based on the pathogenesis, clinical manifestation, and treatment, obesity can be classified into two broad types, namely, “shi obesity” and “xu obesity.” The aim of this study was to explore the differences in metabolite levels between these two types of obesity.

**Methods:**

Q Exactive liquid chromatography–tandem mass spectrometry was used to analyze the metabolites in the sera of 30 healthy adults, 30 adults with shi obesity, and 30 adults with xu obesity. Possible metabolic pathways associated with biological alterations were identified using the Human Metabolome Database and Kyoto Encyclopedia of Genes and Genomes analyses.

**Results:**

Fifteen important potential biomarkers were identified, 14 of which were upregulated. Furthermore, the biomarkers phenylalanine, L-tyrosine, glycerophosphocholine, and LysoPC(O-18:0/0:0) were significantly correlated with both shi and xu obesity. Of note, L-tyrosine and glycerophosphocholine were screened and used as potential biomarkers of shi and xu obesity for the first time.

**Conclusion:**

This study verified the differences in serum metabolites associated with shi and xu obesity, demonstrating potential therapeutic strategies for the two types.

## Background

Obesity is one of the most prevalent health issues in modern civilization, and the global obesity levels have increased threefold since 1975. Among adults over the age of 35 years, 13% are obese and 39% are overweight [1]. Obesity can cause numerous metabolic disorders, including hyperlipidemia, nonalcoholic fatty liver disease, hypertension, type 2 diabetes, and cardiovascular diseases [2]. Furthermore, obesity has been linked to stroke, several malignancies, and arthritic conditions [3]. Therefore, obesity poses a serious risk to human health. Obesity results from prolonged energy imbalance, genetic susceptibility, and environmental factors [4], leading to pathological fat accumulation. The primary symptom of obesity is an increase in plasma levels of free fatty acids, cholesterol, triglycerides, total lipids, and other lipid components. Obesity is a complicated multifactorial illness influenced by several factors, including genetics, living conditions, and lifestyle choices. The treatment is complicated by individual heterogeneity and the absence of a scientific warning system. Modern medicine asserts that the health damage caused by obesity is related not only to the degree of obesity and fat content but also to the ectopic accumulation of fat in the body [5]. According to the World Health Organization classification criteria, obesity is defined in terms of the body mass index, which is calculated as weight in kilograms divided by the square of height in meters [6].

In traditional Chinese medicine (TCM), obesity is defined as the accumulation of fat and unmetabolized nutrients in the body that causes an increase in body weight and various symptoms such as exhaustion, weakness, and shortness of breath [7]. This disease occurs in the spleen and stomach, and its basic pathogenesis involves phlegm and dampness after mid-fullness, leading to qi stagnation, blood stasis, and internal heat [8]. As early as the *Huangdi Canon of Medicine* categorized obese individuals into three types: those who are fat and have loose flesh, those who are fat and elastic, and those who are fat and muscular. In essence, the distribution of fat is considered the main diagnostic principle. Considering the differences in constitution and clinical manifestations, Chinese medicine further classifies this syndrome into “shi obesity” and “xu obesity”. Obese individuals who are fat or fat and muscular fall in the shi obesity group, and those who are fat and have loose flesh fall in the xu obesity group, which must be treated according to the evidence typing [9]. Shi obesity is characterized by waist-to-hip ratio (WHR)<1, firm abdomen, strong appetite, and normal metabolic hormones; Xu obesity by WHR>1, flabby abdomen, fatigue, and low metabolic rate [10,11]. Diagnostic criteria align with TCM guidelines (ref: Guidelines for Clinical Research of New Chinese Medicines, 2002 [12])”.

Shi obesity, generally observed in young adults, is mostly peripheral and metabolically and typically manifests as normal metabolic indicators and hormone secretion [13]. Individuals with shi obesity have a well-proportioned body with a waist-to-hip ratio of < 1, a strong appetite, a firm abdomen, thick hair, and sticky or dry stools. Shi obesity is caused by excessive food intake leading to phlegm and dampness accumulation in the body, which cannot be quickly consumed, resulting in fat formation and obesity. The main treatment is the combined clearing-purging method [14]. Xu obesity is predominantly abdominal and low metabolic obesity and is characterized by a low metabolic rate, low hormone levels, and a waist-to-hip ratio > 1. Most patients with xu obesity are weak, lack strength in the waist and knees, have a flabby abdomen, are bloated, have disproportionate weight distribution, and are less energetic [10]. Most individuals present with physical weakness, fatigue, and abdominal laxity. Therefore, brewing dampness generates phlegm, which in turn leads to obesity, and treatment is mostly based on invigorating the spleen [9].

Metabolite-level investigations of obesity have identified the biomarkers of distinct forms of obesity. Investigating the metabolic distinctions between xu and shi obesity is critical for predicting and diagnosing both these obesity syndromes. Over the past decade, metabolomics has expanded rapidly, becoming a potent tool for understanding and forecasting complicated phenotypes in a wide variety of biological systems [15]. Furthermore, because of gene expression, metabolomics has been extensively employed in the medical field to elucidate how medications or the disease itself affects certain metabolic processes and to identify potential biomarkers for characterizing physiological and pathological endpoints [16]. As serum is a readily available biofluid [17], its metabolic profile is useful for characterizing both healthy and pathological conditions and distinguishing between distinct disease markers at the metabolic level. Serum metabolomics has been extensively employed in research on the pathophysiology of obesity-related disorders, including diabetes mellitus and hyperlipidemia [18], as well as variations in associated biomarkers following various medication regimens.

Currently, evidence for serum metabolomic biomarkers related to shi and xu obesity remains insufficient. Therefore, in this study, metabolomics was used to characterize the differences in metabolic levels between adults with shi and xu obesity. The findings can be used to identify the biomarkers of the two types of obesity.

## Materials and methods

### Sample source

The samples were sourced from the Preventive Treatment Center of Changchun University of Traditional Chinese Medicine. Subjects were recruited according to the diagnostic criteria for TCM evidence elements of obesity. The subjects were scored according to the clinical symptoms within the requirements, with a total score of <9 categorized as shi obesity and a total score of ≥9 categorized as xu obesity. Ninety subjects were recruited, of whom 30 were healthy, 30 were in the solid fat group, and 30 were in the deficient fat group [19]. Symptoms/scores: Based on TCM evidence criteria (fat distribution, appetite, abdominal firmness, fatigue; score<9: Shi, ≥ 9:Xu) [12]. Scores combined TCM symptoms (e.g., fatigue, abdominal firmness); detailed in Suppl. Table 1. Group labels: Shi obesity (n = 30, WHR < 1), Xu obesity (n = 30, WHR > 1). Gender: 53% female (n = 48), 47% male (n = 42), matched across groups.

**Table 1:** Baseline demographic and clinical characteristics (mean + SD). Waist-to-Hip Ratio (WHR):Shi < 1, Xu > 1 (P < 0.01).

Variable	Healthy group	Xu obesity group	Shi obesity group
Age, years	27.0 ± 5.0	27.1 ± 6.4	25.4 ± 2.6
BMI	22.8 ± 2.4	30.5 ± 3.9	30.4 ± 3.6
High density lipoprotein	1.3 ± 0.3	1.3 ± 0.3	1.3 ± 0.2
Low density lipoprotein	2.9 ± 0.7	3.1 ± 1.0	3.0 ± 0.6
Triglycerides	1.4 ± 0.7	1.8 ± 1.5	1.7 ± 1.6
Total cholesterol	4.7 ± 0.8	5.0 ± 1.1	4.9 ± 0.8

This study complied with all the ethical statements of the Declaration of Helsinki and was approved by the Ethics Committee of Changchun University of Traditional Chinese Medicine (CCZYFYKYLL2023). The methods and objectives were explained to the eligible subjects, and written informed consent was obtained before participation. The recruitment started on October 20, 2023, and ended on November 20, 2023.

Based on expert experience and the third and tenth editions of *Internal Medicine of Traditional Chinese Medicine* published by the China Medical Science and Technology Press, along with clinically developed standards, the 2002 edition of *Guidelines for Clinical Research of New Chinese Medicines* [12] was used in this study. People with a large amount of fat and a hard front, as well as fat, muscular people, were categorized into the shi obesity group, which was further divided into stomach heat syndrome and phlegm dampness syndrome. People with large amounts of fluffy fat were categorized into the xu obesity group, which was further divided into spleen depletion and yang deficiency in the spleen and kidneys. “Fluffy fat” was defined as loose, non-firm subcutaneous adipose tissue palpated clinically.

### Sample preparation

10 mL of venous blood was collected, clotted at 25°C for 30 min, and centrifuged at 3,000 rpm for 15 min to isolate serum. To prepare samples for analysis, samples were thawed at 4 °C, after which 250 µL of serum was mixed with 750 µL of pre-cooled methanol to precipitate proteins and vortex for 1 min. The mixture was centrifuged at 12,000 rpm for 15 min at 4 °C, the supernatant was transferred to a new centrifuge tube, and the sample was blown dry with nitrogen. Thereafter, 800 µL of pre-cooled methanol was added, and the mixture was vortexed and mixed. After centrifugation at 12,000 rpm for 15 min, the supernatant was filtered through a 0.22 µm membrane filter for liquid chromatography–tandem mass spectrometry (LC-MS/MS) analysis. From each sample, 10 µL was used as a quality control (QC) sample to test the instrument stability and data reproducibility.

### Q Exactive LC-MS/MS analysis of serum

Serum samples from 30 healthy adults, 30 adults with shi obesity, and 30 adults with xu obesity were analyzed using Q Exactive LC-MS/MS. Potential biomarkers were screened by comparing metabolites with significantly different abundances among the three groups and combining them with the corresponding pathway enrichment results.

LC separation was performed on a Thermo UltiMate 3000 instrument with a Supelco Ascentis Express C18 column (50 mm × 3.0 mm, 2.7 µm, Sigma‒Aldrich). The column temperature was maintained at 40 °C, the flow rate was 0.4 mL/min, and the injection volume was 5 µL. The following gradient elution program, with 0.1% (*v/v*) formic acid in water as mobile phase B and 100% (*v/v*) acetonitrile as mobile phase C, was used in both positive and negative ion modes: 10%–20% C (*v/v*), 0–3 min; 20%–40% C (*v/v*), 3–5 min; 40%–60% C (*v/v*), 5–7 min; 60%–80% C (*v/v*), 7–9 min; 80%–90% C (*v/v*), 9–11 min; 90%–95% C (*v/v*), 11–20 min; 95%–5% C (*v/v*), 20–22 min; and 5%–5%(*v/v*), 22–25 min.

This LC system was integrated with a Thermo Q Exactive mass spectrometer equipped with an electrospray ionization source and operated simultaneously in the positive and negative ion modes. In both modes, the spray voltage was 2.5 kV, the flow velocities of the sheath and auxiliary gases were set at 40 and 10 arbitrary units, respectively, and the auxiliary gas temperature was 300 °C. The mass scan range was set to *m*/*z* 50.0–2000.0 for full scans, and the mass resolution was 70,000.

### Data processing and multivariate statistical analysis

The serum sample data were processed using Sieve (version 2.1, Thermo Fisher Scientific, USA) for peak extraction, alignment, and normalization. Data pre-processing produced a numerical matrix containing the sample code, *m/z*, retention time, and peak intensity. The peak data extracted from all experimental and QC samples were imported into MetaboAnalyst 5.0 (https://www.metaboanalyst.ca/) for sparse partial least squares discriminant analysis (sPLS-DA), which allowed for variable selection and the identification of predictive or discriminatory features in the data for sample classification. The outliers were removed to improve the accuracy of the model. To describe the differences in serum metabolic profiles between the healthy, shi obesity, and xu obesity groups, the peak data extracted from all experimental samples were imported into Simca-*P* 14.0 for orthogonal partial least squares discriminant analysis (OPLS-DA). This approach was subsequently used for supervised pattern recognition of the serum data to identify the differences between the groups. A random permutation test was performed 200 times on the data, and the accuracy of the mathematical model was evaluated based on the R^2^ and Q^2^ values. Changes in serum metabolites in each group were subjected to multifactorial statistical analysis and further screened for differential metabolites based on the significance of variables in the OPLS-DA model projection (VIP > 1) and Student’s t-test (**P* *< 0.05).

The compounds detected by LC-MS/MS were identified by searching for accurate masses of the peak features against the Kyoto Encyclopedia of Genes and Genomes (KEGG) database (http://www.kegg.jp/) and Human Metabolome Database (HMDB) (http://www.hmdb.ca). Finally, we retrieved the endogenous compounds corresponding to each *m/z* ratio to obtain metabolic information. MetaboAnalyst 5.0 (https://www.metaboanalyst.ca/) was used for additional enrichment and pathway analyses of previously identified potential metabolites.

## Results

### General characteristics of the study participants at recruitment

The table below shows the study participants’ anthropometric, physiological, and biochemical characteristics during recruitment (Table 1).

### Method validation

**Fig 1:**
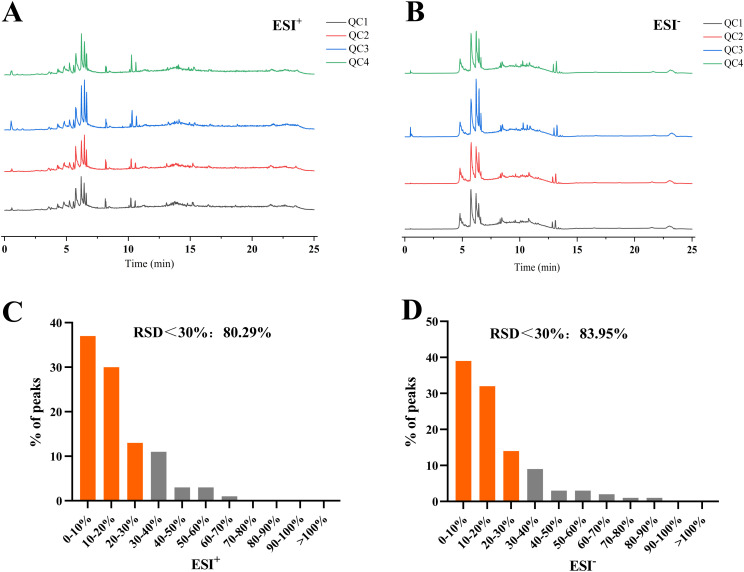
Total ion chromatograms of QC samples in positive (A) and negative (B) ion modes; proportions of characteristic peaks with relative standard deviation (RSD) of less than 30% in quality control (QC) samples in positive (C) and negative (D) ion modes.

This method was validated prior to sample testing. The QC sample was analyzed every tenth sample throughout the acquisition process to ensure instrument stability and data reliability. QC samples can correct signals, thereby reducing analytical variation and quantitatively determining analytical precision. As shown in Fig 1A and Fig 1B, the total ion current chromatograms of the QC samples were compared by overlaying the spectra. The response intensity and retention time of each chromatographic peak overlapped, indicating that a small variation was caused by instrumental errors throughout the experimental process. A smaller relative standard deviation (RSD) for the characteristic peak abundance of QC samples corresponds to better instrument stability. Therefore, features with poor repeatability in the QC samples were removed, and sample data with an RSD of less than 30% were retained for a higher-quality dataset that would facilitate the detection of potential biomarkers. As shown in Fig 1C and Fig 1D, the proportion of characteristic peaks in the QC samples with an RSD of less than 30% in the positive and negative ion modes reached 80.29% and 83.95%, respectively, indicating that the data were reliable and could be used for subsequent analysis.

### Multivariate statistical analysis based on Q Exactive LC-MS/MS

**Fig 2:**
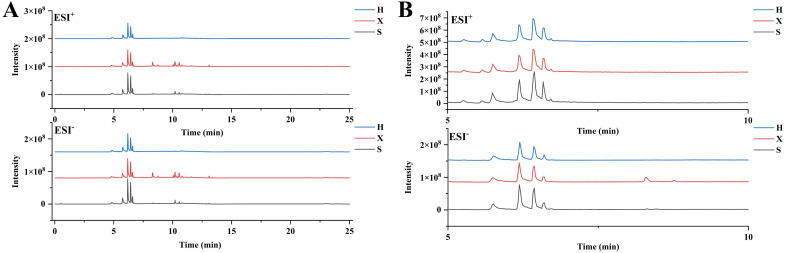
Overlaid total ion current (TIC) curves (A), zoomed region (RT 5–10 min) highlighting key peaks (B).

Fig 2 shows the total ion chromatograms of the serum samples from the healthy, shi obesity, and xu obesity groups. The peak areas and retention times in the shi obesity group were significantly different from those in the xu obesity group. Therefore, metabolites with obvious differences in content were monitored. Multivariate statistical analysis was conducted to visualize chemical differences among the three groups.

**Fig 3:**
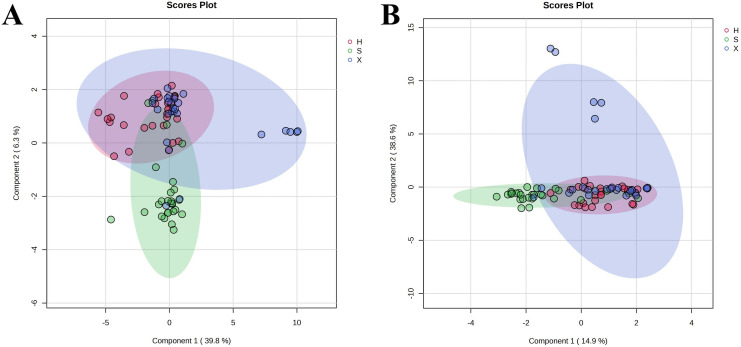
sPLS-DA scores of the healthy, shi obesity, and xu obesity groups (healthy group: H – red; shi obesity group: S – green; xu obesity group: X – blue) in positive (A) and negative (B) ion modes.

The sPLS-DA algorithm reduces the number of variables (in this case, metabolites) to produce robust and easy-to-interpret models. In this study, the sPLS-DA model was used to analyze the changes in the serum metabolite content in the three groups, which displayed a trend toward separation. As shown in Fig 3A and Fig 3B, the distributions within the three groups were relatively clustered. Although the groups exhibited some overlap, a clear separation trend was observed, demonstrating differences in the metabolic levels of the three groups.

**Fig 4:**
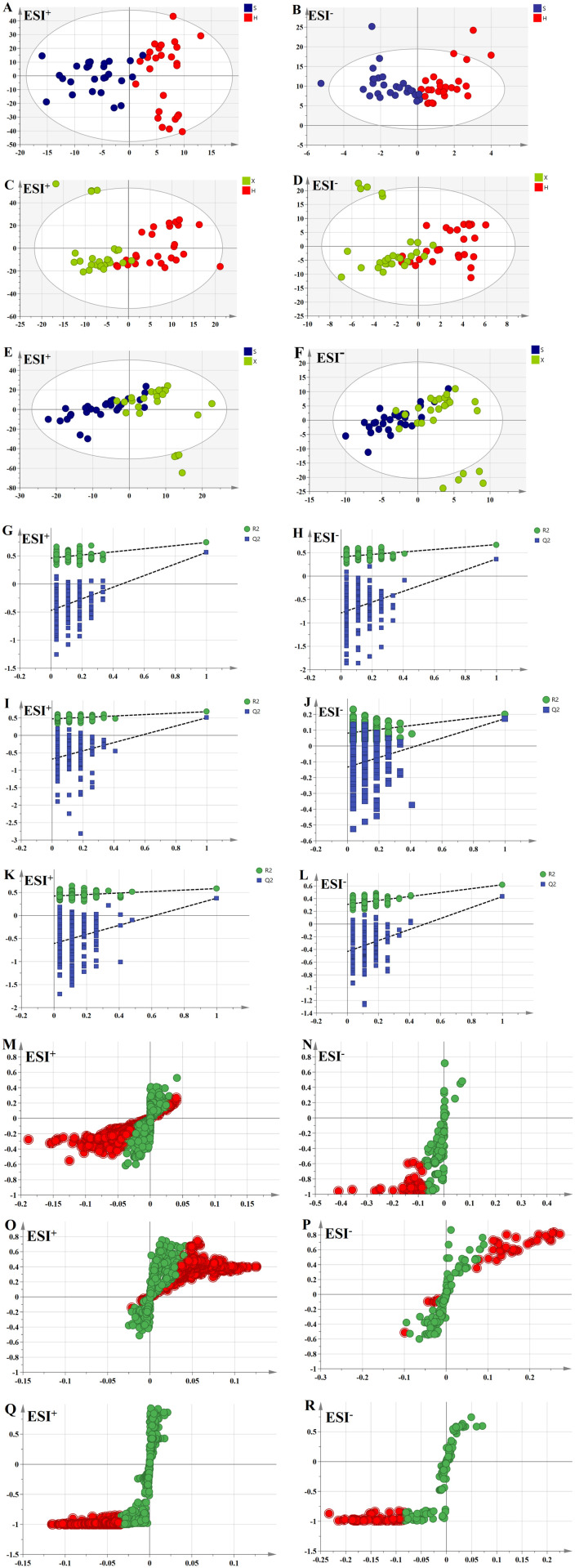
Multivariate statistical analysis. OPLS-DA scores of each control group in positive and negative ion modes: healthy group vs. shi obesity group **(A, B)**, healthy group vs. xu obesity group **(C, D)**, and shi obesity group vs. xu obesity group **(E, F)** (healthy group: H – red; shi obesity group: S – blue; xu obesity group: X – green). Two hundred displacement test charts for each control group in positive and negative ion modes: healthy group vs. shi obesity group **(G, H)**, healthy group vs. xu obesity group **(I, J)**, and shi obesity group vs. xu obesity group **(K, L)**. S-plots for each control group in positive and negative ion modes: healthy group vs. shi obesity group **(M, N)**, healthy group vs. xu obesity group **(O, P)**, and shi obesity group vs. xu obesity group **(Q, R)** (red circles indicate metabolites with VIP ≥ 1).

To extract further information regarding the differences between the three groups, supervised OPLS-DA was performed on the three datasets. In the model of the healthy group and the shi obesity group, the R^2^Y and Q^2^ values under the established OPLS-DA model were 0.740 and 0.564, respectively, in the positive ion mode and 0.674 and 0.367, respectively, in the negative ion mode. In the model of the healthy group and the xu obesity group, the R^2^Y and Q^2^ values under the established OPLS-DA model were 0.679 and 0.506, respectively, in the positive ion mode and 0.618 and 0.432, respectively, in the negative ion mode. In the model of the shi obesity group and the xu obesity group, the R^2^Y and Q^2^ values under the established OPLS-DA model were 0.584 and 0.376, respectively, in the positive ion mode and 0.526 and 0.282, respectively, in the negative ion mode. In both positive and negative ion modes, there was a clear separation or separation trend between the healthy group and the shi obesity group (Fig 4A and Fig 4B), the healthy group and the xu obesity group (Fig 4C and Fig 4D), and the shi obesity group and the xu obesity group (Fig 4E and Fig 4F), indicating the existence of differences in metabolites among the three groups.

S-plots were generated to search for endogenous, differentially abundant metabolites between the groups for the healthy and shi obesity groups (Fig 4M and Fig 4N), for the healthy and xu obese groups (Fig 4O and Fig 4P), and for the shi and xu obesity groups (Fig 4Q and Fig 4R). Metabolites located closer to the upper right and lower left corners exhibit more significant differences, and red circles indicate metabolites with VIP values of ≥1. For model validation, model data were subjected to multiple randomized permutation experiments (*n* = 200). Notably, for the models established to compare the healthy and shi obesity groups (Fig 4G and Fig 4H), healthy and xu obesity groups (Fig 4I and Fig 4J), and shi and xu obesity groups (Fig 4K and Fig 4L), the intercepts of the Q2 regression lines on the vertical axis are less than 0 for all three control groups, indicating that the models have good predictive ability and are not overfitted, thereby confirming their dependability and consistency [20].

### Selection and identification of biomarkers

Using **P* *≤ 0.05 and VIP ≥ 1, we screened potential biomarkers. 1,206 metabolites were detected; 529 were differentially abundant (shi vs. xu). A total of 184 differentially abundant metabolites were identified between the healthy and shi obesity groups, 307 differentially abundant metabolites were identified between the healthy and xu obesity groups, and 529 differentially abundant metabolites were identified between the shi and xu obesity groups.

To determine potential metabolic biomarkers for the healthy and shi obesity groups, 15 metabolites (Table 2) among the differentially abundant metabolites were screened based on the fold-change threshold (FC (Fold Change) > 1.5 or FC < 0.667) of the metabolite expression level [20]. Specifically, the screened metabolites included one between the healthy and shi obesity groups, nine between the healthy and xu obesity groups, and 12 between the shi and xu obesity groups. Based on the precise molecular weights of the differentially abundant metabolites, accurate metabolite information was obtained using the HMDB (http://www.hmdb.ca) and KEGG databases (http://www.kegg.jp/).

**Table 2:** Potential biomarkers in healthy individuals and those with different obesity syndromes. (VIP ≥ 1, P < 0.05).

Identified metabolites	Formula	Detected *m/z*	Adducts	HMDB ID	H/S		H/X		S/X	
					VIP	Trend	VIP	Trend	VIP	Trend
L-Carnitine	C_7_H_15_NO_3_	162.1152	[M+H]^+^	HMDB0000062	—	—	—	—	1.23649	↓^**^
Phenylalanine	C_9_H_11_NO_2_	166.0866	[M+H]^+^	HMDB0000159	—	—	1.06743	↓^*^	—	—
L-Tyrosine	C_9_H_11_NO_3_	182.0841	[M+H]^+^	HMDB0000158	—	—	—	—	1.24903	↓^**^
3,4,5,6-Tetrahydrohippuric acid	C_9_H_13_NO_3_	184.0975	[M+H]^+^	HMDB0061679	—	—	—	—	1.23234	↓^**^
Glycerophosphocholine	C_8_H_20_NO_6_P	258.1142	[M+H]^+^	HMDB0000086	—	—	1.24689	↓^*^	1.40277	↓^**^
Methionyl-Methionine	C_10_H_20_N_2_O_3_S_2_	281.0997	[M+H]^+^	HMDB0028979	—	—	1.23493	↓^*^	1.33555	↓^**^
5-Aminoimidazole ribonucleotide	C_8_H_14_N_3_O_7_P	296.0701	[M+H]^+^	HMDB0001235	—	—	1.34318	↓^*^	1.40601	↓^**^
Dodec-7-enedioylcarnitine	C_19_H_33_NO_6_	313.1588	[M+H]^+^	HMDB0241228	—	—	—	—	1.02603	↓^*^
LysoPE(P-16:0/0:0)	C_21_H_44_NO_6_P	438.3045	[M+H]^+^	HMDB0011152	—	—	—	—	1.11619	↓^*^
LysoPC(P-16:0/0:0)	C_24_H_50_NO_6_P	480.3526	[M+H]^+^	HMDB0010407	—	—	1.15207	↓^*^	1.13416	↓^*^
LysoPC(P-18:1(9Z)/0:0)	C_26_H_52_NO_6_P	506.3685	[M+H]^+^	HMDB0010408	—	—	1.20103	↓^*^	1.13257	↓^*^
LysoPC(P-18:0/0:0)	C_26_H_54_NO_6_P	508.3839	[M+H]^+^	HMDB0013122	—	—	1.23242	↓^*^	1.15753	↓^*^
LysoPC(O-18:0/0:0)	C_26_H_56_NO_6_P	510.3996	[M+H]^+^	HMDB0011149	—	—	1.22554	↓^**^	—	—
Isolariciresinol glucuronide	C_26_H_32_O_12_	537.2026	[M+H]^+^	HMDB0240735	—	—	—	—	1.20004	↓^**^
TG(a-25:0/i-24:0/i-20:0)	C_72_H_140_O_6_	1102.0791	[M+H]^+^	HMDB0070953	1.69034	↑^**^	—	—	—	—

*p < 0.05.

**p < 0.01.

***p < 0.001 (healthy group: H; shi obesity group: S; xu obesity group: X)

**Fig 5:**
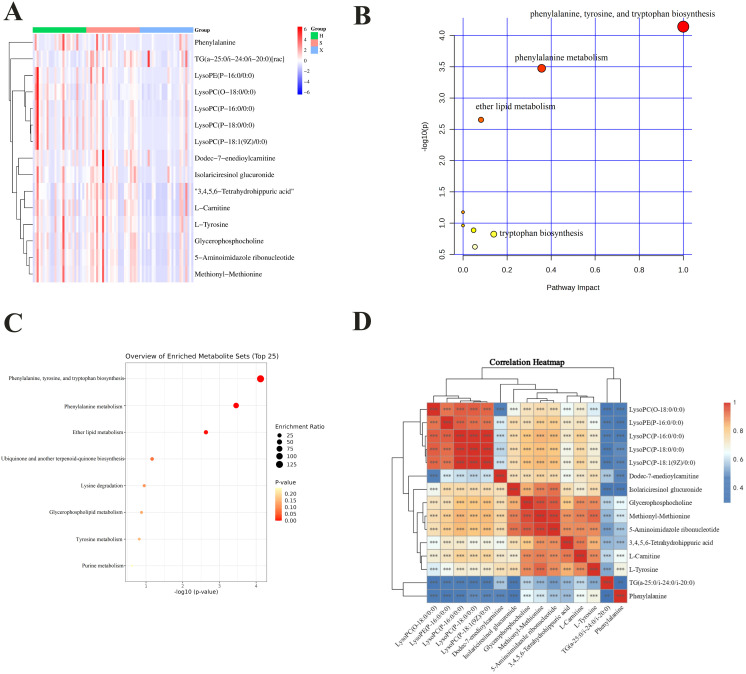
Differentially abundant metabolite cluster analysis heat map (A). Pathway analysis of differentially abundant metabolites **(B)**. Enrichment analysis of differentially abundant metabolites **(C)**. Correlation heatmap of differentially abundant metabolites **(D)** (red and blue indicate positive and negative correlation, respectively).

Considering the criteria that log2FC ≥ 1 indicates that a metabolite is upregulated and log2FC ≤ −1 indicates that a metabolite is downregulated, log2FC values were calculated [21]. Compared to the healthy group, one upregulated marker was found in the shi obesity group, and nine downregulated markers were found in the xu obesity group. Compared to the shi obesity group, the xu obesity group contained 12 downregulated markers. MetaboAnalyst 5.0 (https://www.metaboanalyst.ca/) was used to compare changes in differentially abundant metabolites among the groups. To further examine the relationships between metabolite abundances among the groups, the relative levels of the potential biomarkers were characterized using a heatmap. As shown in Fig 5A, clustering analysis revealed changes in the differentially abundant metabolites in the healthy group compared to those in the shi and xu obesity groups, as well as a trend toward changing metabolite concentrations. Color differences between the three groups indicate the presence of metabolite differences in the shi and xu obesity groups, with metabolites clustered in the same group having similar expression patterns.

### Analysis of metabolic pathways

The authors mention that 15 metabolites were used for further analysis, but there are more than 15 metabolites in Table 2. To explore the significant differentially abundant metabolites, metabolic pathways, and metabolic mechanisms among the three groups, the 15 selected metabolites were imported into MetaboAnalyst 5.0 for metabolic pathway enrichment analysis (Fig 5B and Fig 5C). As shown in Fig 5B, the 15 biomarkers participated in eight related metabolic pathways: phenylalanine, tyrosine, tryptophan biosynthesis; phenylalanine metabolism; ether lipid metabolism; ubiquinone and other terpenoid-quinone biosynthesis; lysine degradation; glycerophospholipid metabolism; tyrosine metabolism; and purine metabolism. Phenylalanine and tyrosine→phenylalanine/tyrosine biosynthesis; glycerophosphocholine/LysoPC→ether lipid metabolism.

Based on the impact and *P* values, the three most important metabolic pathways were considered to be those most closely associated with xu and shi obesity, namely, phenylalanine, tyrosine, and tryptophan biosynthesis; phenylalanine metabolism; and ether lipid metabolism (Table 3). Thus, individuals with shi or xu obesity may develop obesity due to disorders in these three metabolic processes. Metabolites enriched in these pathways, including phenylalanine, L-tyrosine, glycerophosphocholine, and LysoPC(O-18:0/0:0), were significantly downregulated (Table 2). Notably, phenylalanine, glycerophosphocholine, and LysoPC(O-18:0/0:0) were downregulated in the xu obesity group compared with the healthy group, whereas L-tyrosine and glycerophosphocholine were downregulated in the xu obesity group compared with the shi obesity group (Table 2).

**Table 3:** Three important related metabolic pathways in healthy individuals and those with different obesity syndromes.

Pathway	KEGG	*P*	Impact	Metabolites	Control group	Trend
Phenylalanine, tyrosine, and tryptophan biosynthesis	hsa00400	7.2362E-5	1.0	Phenylalanine	H/S	Down
				L-Tyrosine	S/X	Down
Phenylalanine metabolism	hsa00360	3.3541E-4	0.35714	Phenylalanine	H/S	Down
				L-Tyrosine	S/X	Down
Ether lipid metabolism	hsa00565	0.00223	0.08176	Glycerophosphocholine	H/S	Down
					S/X	Down
				LysoPC(O-18:0/0:0)	H/X	Down

(healthy group: H; shi obesity group: S; xu obesity group: X)

### Correlation analysis of potential metabolic biomarkers

The pathway analysis results showed that significant downregulation of phenylalanine and L-tyrosine led to disorders in phenylalanine, tyrosine, and tryptophan biosynthesis and phenylalanine metabolism, whereas significant downregulation of glycerophosphocholine and LysoPC(O-18:0/0:0) caused ether lipid metabolism disorders.

After combining multiple factors, 15 significantly differentially abundant metabolites were subjected to correlation analysis, and the results are visually presented as a correlation heatmap (Fig 5D). While multiple metabolites correlated (Fig 5D), Phe-Tyr synergy reflects shared aromatic amino acid metabolism, implicated in insulin resistance [22], underscoring their biological significance [23]. The correlation heatmap revealed a positive correlation between phenylalanine and L-tyrosine, which are involved in the same pathway. Although L-tyrosine and glycerophosphocholine are involved in different metabolic pathways, they are strongly correlated. Thus, among the 15 biomarkers for shi and xu obesity, L-tyrosine and glycerophosphocholine require further investigation.

## Discussion

As a chronic illness, obesity has become a significant global public health concern. Metabolomic analysis has been empirically established as a promising research methodology for studying TCM syndromes [24]. Additionally, serum, an accessible and information-dense biological fluid, comprehensively reflects the *in vivo* metabolic effects of diseases and medications.

A review of previous studies revealed that phlegm-damp and qi-deficient individuals in the obese population have greater lipoprotein content but lower lactate and glycine concentrations than individuals in the normal population [21]. Metabolomic analysis of rats with spleen deficiency has revealed that 13 small-molecule fatty acid metabolites, including arachidonic acid, 2-hydroxybutyric acid, oleic acid, and palmitic acid, contribute to the disruption of glucose and lipid metabolism [25]. Using serum samples from a healthy group and a phlegm syndrome spleen group, 230 metabolites were compared. Among the 27 specific metabolites identified, amino acids and free fatty acids were predominant and biotin metabolism was the main pathway of influence. Moreover, lipid, amino acid, and glycerophospholipid metabolism disorders were prevalent in patients with phlegm syndrome [26].

In the present study, metabolomic analysis was used to screen 15 potential biomarkers. These biomarkers primarily disrupt metabolic pathways, including phenylalanine metabolism, tyrosine and tryptophan biosynthesis, and ether lipid metabolism. Notably, phenylalanine, L-tyrosine, glycerophosphocholine, and LysoPC (O-18:0/0:0) were found to be associated with the development of obesity. The levels of phenylalanine, glycerophosphocholine, and LysoPC(O-18:0/0:0) were significantly lower in the xu obesity group than in the healthy group, whereas the levels of L-tyrosine and glycerophosphocholine were significantly lower in the xu obesity group than in the shi obesity group. These four compounds were identified as differentially abundant metabolites that participate in the three most important metabolic pathways related to obesity.

Obesity can trigger metabolic disorders in the body, which are often accompanied by changes in related biomarkers. Common metabolic markers include amino acids, lipids, phospholipids, fatty acids, choline, and carnitine [27]. Our findings indicate that shi and xu obesity are mostly associated with amino acid metabolism (disrupting phenylalanine, tyrosine, and tryptophan biosynthesis and phenylalanine metabolism) and ether lipid metabolism. Downregulation of glycerophosphocholine and LysoPC(O-18:0/0:0) disrupts ether lipid metabolism, which is implicated in cardiovascular disease and hepatic steatosis [28,29]. Similarly, phenylalanine/tyrosine imbalances correlate with insulin resistance and diabetes risk [23,30], suggesting shared pathways between TCM obesity subtypes and metabolic syndromes. While conventional obesity classifications focus on BMI and cardiometabolic risk [31], Shi/Xu subtypes reflect TCM pathophysiology. Metabolites like glycerophosphocholine (downregulated in Xu) align with ‘metabolically unhealthy obesity’ in Western medicine, characterized by lipid dysregulation [5,32]. This bridges TCM subtypes with established metabolic phenotypes.

Glycerophosphocholine is involved in ether lipid and glycerophospholipid metabolism, which can significantly increase the risk of conditions such as atherosclerosis, thereby influencing the incidence of cardiovascular disease [29]. Choline is an important component in the synthesis of low-density lipoproteins, which is the only carrier in the liver that transports fat to the periphery. Low-density lipoprotein can also transport triglycerides to the liver and prevent their deposition. Obesity plays a key role in promoting cardiovascular disease, certain types of cancer, type 2 diabetes, and nonalcoholic fatty liver disease through inflammation [32], abnormal adipokine release, and increased free fatty acid levels. Nonalcoholic steatohepatitis and hepatic steatosis are associated with the downregulation of glycerophosphocholine [28]. Decreased tyrosine and tryptophan levels in the serum of patients with lung cancer can assist in classifying lung cancer tissue types and tumor staging [33]. In addition, glycerophosphocholine, which is closely associated with multiple types of cardiovascular diseases in adolescents, may be a sensitive indicator of the risk of cardiovascular diseases and obesity-related issues in adulthood [34].

We found that individuals with xu obesity may have a greater risk of developing obesity-related diseases than those with shi obesity. Common obesity biomarkers (e.g., branched-chain amino acids [22]) were detected but not differentially abundant between Shi/Xu subtypes. This highlights the specificity of phenylalanine/tyrosine dysregulation to TCM classification [23]. Xu obesity showed greater downregulation of glycerophosphocholine (vs. Shi: FC = 0.399; vs. healthy: FC = 0.452), which regulates lipid transport and is associated with cardiovascular risk [34]. Decreased glycerophosphocholine levels are associated with metabolic syndrome in individuals with splenic deficiency syndrome. Additionally, lipid metabolism disorders are associated with splenic deficiency syndrome [22]. Therefore, glycerophosphocholine is a potential target for the treatment of splenic deficiency syndrome. Subtypes (e.g., spleen-kidney yang deficiency in Xu) were not analyzed due to sample size limitations. Future studies should explore metabolic differences within these subcategories. According to TCM principles, the main pathogenesis of xu obesity is a deficiency in the spleen qi, leading to qi imbalance, internal retention of dampness and turbidity, and accumulation in the body, causing obesity [22]. Based on these factors, this study preliminatively explored glyceryl choline and L-tyrosine as potential markers to distinguish shi obesity from xu obesity. Correlation analysis can help measure the closeness between metabolites with significantly different abundances. Metabolites with correlated expression may jointly participate in certain biological processes (i.e., functional correlations). In addition, synergistic or mutually exclusive relationships existed between differentially abundant metabolites (i.e., positive and negative correlations). Positively correlated metabolites may be derived from the same synthetic pathway, whereas negatively correlated metabolites may be decomposed during the synthesis of other metabolites. Compared with the xu obesity group, glycerophosphocholine was upregulated in the shi obesity group. Elevated glycerophosphocholine occurs in phlegm-dampness syndromes [35], consistent with Shi obesity pathogenesis. The level of glycerophosphocholine in the serum of rats with precancerous gastric lesions is significantly increased [36]. According to TCM principles, the functions of the spleen and stomach, transportation, and metabolism are impaired and endogenous phlegm dampness is generated, which accumulates in the body over time, leading to obesity. This behavior further confirms that glycerophosphocholine is a potential marker for distinguishing shi obesity from xu obesity.

Phenylalanine is an important metabolite that is involved in the oxidation and synthesis of fatty acids. Phenylalanine can be converted into tyrosine and ultimately decomposed into pyruvate and acetyl-CoA [37], which enter the tricarboxylic acid cycle and participate in energy metabolism. The phenylalanine content in the healthy group was higher than that in the xu obesity group, and the content of L-tyrosine content in the shi obesity group was higher than that in the xu obesity group. A prospective study identified a predictive association between aromatic amino acids (e.g., L-tyrosine) and the development of hyperglycemia as well as progression to clinically manifest diabetes [23,30]. A metabolic imbalance between phenylalanine and tyrosine may lead to fat metabolism disorders and increased fat deposition [38]. Therefore, we preliminarily found that these two metabolites could be used as diagnostic indicators of shi obesity and xu obesity. Glycerophosphocholine is a useful source of choline, an essential nutrient required for cell membrane integrity, signaling, and lipid transport [39]. LysoPC(O-18:0/0:0) downregulation in Xu obesity aligns with phospholipid metabolism disorders in splenic deficiency [31], potentially contributing to ectopic lipid deposition [22]. Glycerophosphocholine has many important physiological functions such as cell signal transduction, lipoprotein secretion, and metabolism, as well as functions in the endoplasmic reticulum and mitochondria. Many animal studies have suggested that glycerophosphocholine metabolism disorders can trigger endoplasmic reticulum stress, metabolic syndromes (e.g., obesity, insulin resistance, and dyslipidemia), and phenylalanine metabolism disorders. Ten weeks of glycerophosphocholine administration in KK-Ay mice significantly reduced serum total cholesterol and triglyceride levels [38]. Studies have demonstrated elevated *PPARα* mRNA levels in the hepatic tissue of glycerophosphocholine supplemented mice, which implies that glycerophosphocholine could mediate lipid homeostasis modulation through the activation of *PPARα* downstream targets, including adipose triglyceride lipase (ATGL) [40]. In this study, significant differences in the levels of various phospholipid metabolites (triglycerides, LysoPC, and LysoPE) were detected among the healthy, shi obesity, and xu obesity groups. A previous report found that rats with coronary heart disease caused by qi deficiency and blood stasis syndrome have a phospholipid metabolism disorder [41], which could include ether lipid metabolism, as identified in our study. According to TCM principles, xu obesity is a manifestation of spleen qi deﬁciency, which is consistent with our results. The levels of glycerophosphocholine and LysoPC(O-18:0/0:0), differentially abundant metabolites involved in the ether lipid metabolism pathway, were downregulated in the xu obesity group. Limitations: The sample size included in this study was large (90 cases in total), and human blood samples themselves had significant individual differences (e.g., age, metabolic status, genetic background) as well as potential physiological and environmental fluctuations (e.g., diet, circadian rhythms). Despite matching age/BMI, unmeasured confounders (e.g., gut microbiota) may contribute to inter-individual variation. Validation in larger cohorts is needed. These factors may have caused some samples to deviate from the main clusters in the multivariate analyses, reflecting the complexity of real biological data. However, further studies with a more representative patient cohort are required to confirm these findings, which suggest that ether lipid metabolism disorders lead to xu obesity.

## Conclusion

The metabolic profiles of individuals with shi and xu obesity were evaluated using Q Exactive LC-MS/MS. Four key metabolites (phenylalanine, L-tyrosine, glycerophosphocholine, LysoPC[O-18:0/0:0]) were prioritized due to their roles in three critical pathways related to metabolic diseases. These three important pathways include: phenylalanine/tyrosine biosynthesis, phenylalanine metabolism, and ether lipid metabolism. The initial findings of L-tyrosine and glycerophosphocholine identified them as potential biomarkers of shi and xu obesity. Although L-tyrosine and glycerophosphocholine participate in different pathways, they display synergistic correlation (r = 0.89, Fig 5D), suggesting coordinated disruption in amino acid and phospholipid metabolism, further demonstrating their research value for the prediction and diagnosis of shi and xu obesity.

## Supporting information

S1 Table Diagnostic criteria for syndrome elements of obesity in Traditional Chinese Medicine. (XLSX)

## Abbreviations

BMI: body mass index
WHR: waist-to-hip ratio
TCM: traditional Chinese medicine
LC-MS/MS: liquid chromatography–tandem mass spectrometry
QC: quality control
TIC: total ion current
ESI: electrospray ionization
HMDB: Human Metabolome Database
KEGG: Kyoto Encyclopedia of Genes and Genomes
RSD: relative standard deviation
sPLS-DA: sparse partial least squares discriminant analysis
OPLS-DA: orthogonal partial least squares discriminant analysis
VIP: variable importance in projection
FC: fold-change threshold
